# Mapping Trends and Hotspots Regarding Clinical Research on COVID-19: A Bibliometric Analysis of Global Research

**DOI:** 10.3389/fpubh.2021.713487

**Published:** 2021-08-23

**Authors:** Demeng Xia, Renqi Yao, Sheng Wang, Gaoqi Chen, Yin Wang

**Affiliations:** ^1^Department of Emergency, Changhai Hospital, Naval Medical University, Shanghai, China; ^2^Department of Orthopaedics, The Naval Hospital of Eastern Theater Command of People's Liberation Army of China (PLA), Zhoushan, China; ^3^Translational Medicine Research Center, Fourth Medical Center and Medical Innovation Research Division of the Chinese PLA General Hospital, Beijing, China; ^4^Department of Burn Surgery, Changhai Hospital, Second Military Medical University, Shanghai, China; ^5^Department of Pancreatic Hepatobiliary Surgery, Changhai Hospital, Naval Medical University, Shanghai, China; ^6^Department of Ultrasound, Shanghai Pulmonary Hospital, Tongji University School of Medicine, Shanghai, China

**Keywords:** COVID-19, clinical research, publications, citation frequency, bibliometrics

## Abstract

**Purpose:** The coronavirus disease 2019 (COVID-19) outbreak, which began in December 2019, has not been completely controlled; therefore, COVID-19 has received much attention from countries around the world. Many related clinical studies, such as clinical trials, have been published, but to the knowledge of the authors, there has been no bibliometric analysis of these publications focusing on clinical research studies on COVID-19.

**Methods:** Global publications on COVID-19 from January 2020 to December 2020 were extracted from the Web of Science (WOS) collection database. The VOSviewer software and CiteSpace were employed to perform a bibliometric study. In addition, we obtained information on relevant clinical trials from the website http://clinicaltrials.gov.

**Results:** China published most of the articles in this field and had the highest number of citations and H-index. The *Journal of Medical Virology* published most of the articles related to COVID-19. In terms of institutions, Huazhong University of Science and Technology had the most publications, and Wang, JW received the highest number of citations.

**Conclusion:** The diagnosis, prevention, and prognosis of COVID-19 are still the focus of attention at present. The overall analysis of the disease were identified as the emerging topics from the perspectives of epidemiology and statistics. However, finding an effective treatment remains the focus of clinical trials.

## Introduction

At the end of December 2019, there was a serious outbreak caused by a type of coronavirus in Wuhan, China, which has been the third coronavirus epidemic since the new millennium, after severe acute respiratory syndrome (SARS) and Middle East respiratory syndrome (MERS). Currently, it is known that this outbreak was caused by a new type of coronavirus, severe acute respiratory syndrome coronavirus 2 (SARS-CoV-2) (1, 2), which is highly pathogenic and infectious with unknown dynamics. Similar to SARS and MERS, SARS-CoV-2 can also cause severe clinical manifestations, ranging from mild symptoms (fever, dry cough, and shortness of breath) to fatal disease (e.g., sepsis and acute respiratory failure) (3). The seasonality of SARS-CoV-2 is unknown, and SARS-CoV-2 has an incubation period (normally 2–14 days) longer than that of SARS. In addition, some patients with coronavirus disease 2019 (COVID-19) have no signs of upper respiratory tract infection, abnormal laboratory findings, or early chest x-ray features, which makes it difficult to diagnose and treat COVID-19 in a timely manner (4, 5). Unfortunately, the number of confirmed cases and the number of deaths due to COVID-19 have progressively increased worldwide.

Coronavirus disease 2019 has undoubtedly become a global health threat, especially for people with underlying diseases, and has become the top priority and a great challenge in more than 200 countries (6). To address this global health threat, the scientific community (such as researchers and research institutions), Medical experts such as Nanshan Zhong and Wenliang Li, and institutions, such as Wuhan University School of Medicine and Huazhong University of Science and Technology, are constantly exploring this field. There are 100s of academic articles published every day that are closely related to various aspects of COVID-19, which include its epidemiology, pathology, drugs, and treatment. (7, 8). As a commonly used research method, clinical studies are of great significance in determining the prognosis of diseases, evaluating drugs, and selecting surgical procedures. Similarly, as an important part of clinical studies, clinical trials are defined as studies on specific drugs in patients or healthy volunteers that evaluate the clinical effects and adverse reactions of experimental drugs and, finally, ensure the effectiveness and safety of the experimental drugs. In the process of dealing with COVID-19, it is even more urgent to obtain effective drugs through clinical trials (9, 10). Therefore, these aspects should be given more attention. Of note, the number of scientific articles on COVID-19 doubles every 15 days since the onset of the epidemic; among them, there is a considerable number of clinical studies. Given that, a large amount of scientific output on COVID-19 makes it difficult to accurately locate information in PubMed, Web of Science (WOS), and Scopus databases by keywords.

To facilitate the search for scientific information related to COVID-19, academics have synthesized and simplified research results mainly through comprehensive reviews, bibliometric analyses, and the establishment of a COVID-19 database. Among them is bibliometrics, which refers to a statistical method that quantitatively analyses publications related to a certain subject *via* mathematical methods, with results providing descriptions and visual quantifications. Based on the bibliometric study of the literature and preprint articles on COVID-19, El Hawary et al. (11), and Kambhampati et al. (12), among others, comprehensively analyzed the subjects, authors, and nationalities of COVID-19-related articles published 3 months before the declaration of the pandemic. Similarly, Odone et al. (13) and Deng et al. (14), among others, have been distinguishing the main branches, authors, journals, and collaborative links of COVID-19 articles since December 2019. However, to the knowledge of the authors, there is no bibliometric analysis specifically focusing on COVID-19 clinical studies. Since the COVID-19 pandemic has not been controlled and more knowledge on the clinical research study is needed, it is urgent to conduct a bibliometric analysis of COVID-19 clinical studies.

## Materials and Methods

### Data Sources and Search Strategies

**Figure 1:**
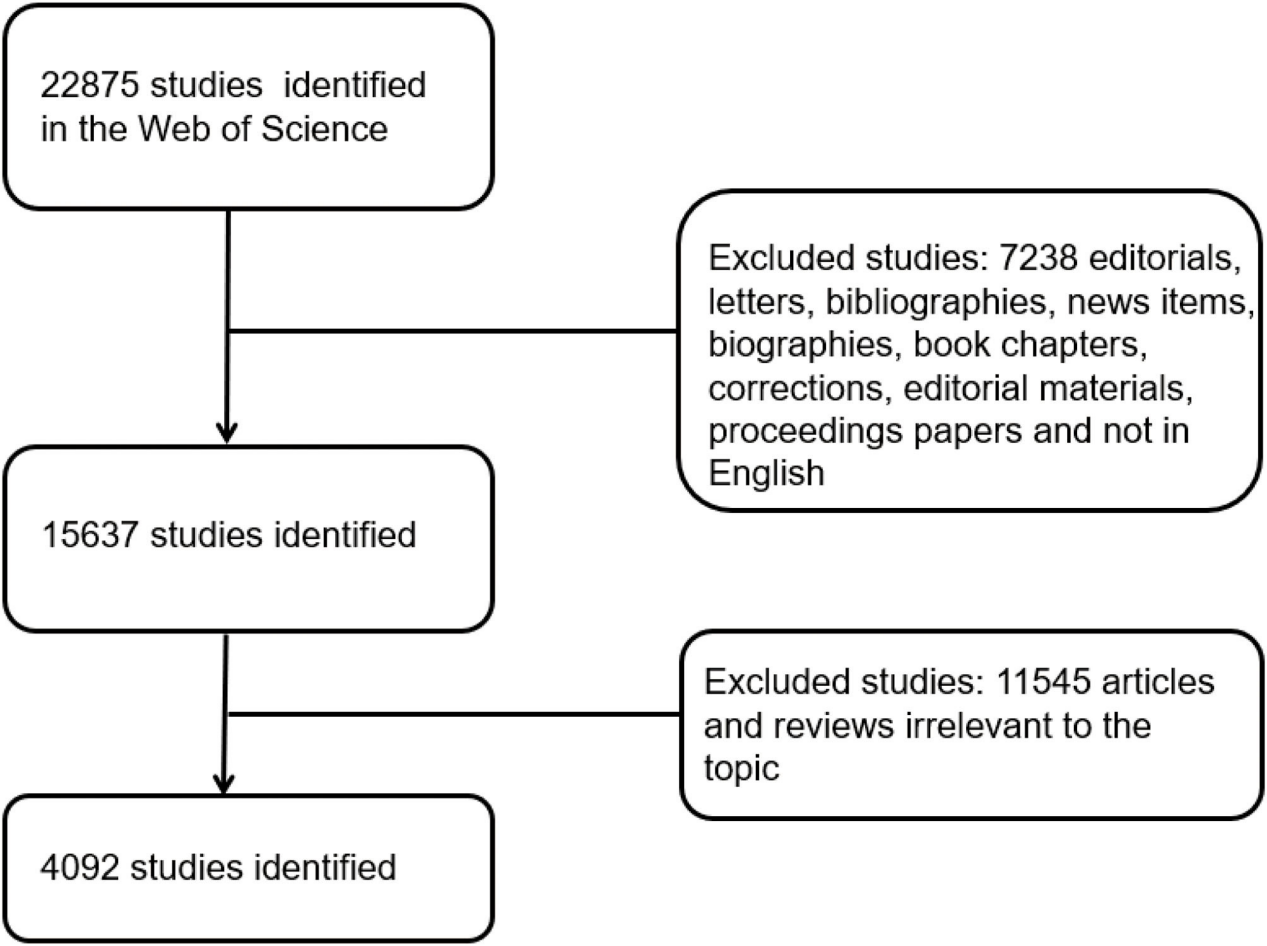
Flow diagram of the inclusion process. The detailed process of screening and enrolment.

The WOS, one of the most appropriate databases for conducting bibliometric analyses, was used to perform comprehensive online searches on clinical studies relevant to COVID-19 from January 2020 to December 2020. As all the data were collected online and no human subjects were involved, ethical consent was not applicable. To avoid bias due to database renewal, we conducted all the searches on December 27, 2020. The search strategy was as follows: TS = (2019 Novel Coronavirus Disease or COVID-19 or coronavirus 2 or SARS-CoV-2 or Novel coronavirus pneumonia or NCP or Novel Coronavirus or 2019-nCoV or coronavirus disease 2019 or coronavirus disease-19) and (Clinical or Trial or Random^*^ or Characteristics or Features or Manifestations or Retrospective or Prospective or Epidemiology or Epidemiologic or Observational or Interventional or Cross-sectional or Case-control or Cohort or Real world or Descriptive). Only original articles and reviews were included in this analysis, and other types of studies were excluded. The search processes were carried out by two of the authors, and if there was a disagreement on inclusion, the final decision was made by the experienced corresponding author. In terms of details, we will first make a preliminary judgment on whether the articles are consistent with the research topic according to the abstract. For the articles that are not certain, we will download the full text and conduct a more detailed evaluation. For the not certain artiles, we will download the full text and conduct a more detailed evaluation. If there are questions, experienced corresponding authors will review them. Detailed information on enrolment and selection is shown in Figure 1. ClinicalTrials.gov is a web-based resource that provides patients and their family members, healthcare professionals, researchers, and the public with easy access to information on publicly and privately supported clinical studies on a wide range of diseases and conditions. Regarding clinical trials, information was obtained from the website http://clinicaltrials.gov. On this website, the search process included the keyword “COVID-19,” and the limitations were “completed” and “clinical trials” (ongoing, stopped halfway, and non-clinical trials were excluded).

### Data Collection and Processing

All the data were extracted from the identified publications by three authors (XDM, YRQ, and WS). The extracted data included titles, keywords, authors, publication dates, countries and regions of origin, institutions, journals, numbers of citations, H-indexes, and other information on publications. Microsoft Excel 2016 (Microsoft, Redmond, WA, United States), GraphPad Prism 8 (GraphPad Prism Software Inc., San Diego, CA, United States), VOSviewer version 1.6.12 (Leiden University, Leiden, the Netherlands), CiteSpace version 5.6.R5 64 bit (Drexel University, Philadelphia, PA, United States), and the Online Analysis Platform of Literature Metrology (http://bibliometric.com/) were applied for the presentation, analysis, and description of the data. Excel is one of the commonly used software for data processing, and it is used to perform a visual representation of histograms in this study. VOSviewer is a program developed for constructing and viewing bibliometric maps of authors or journals based on co-citation data or constructing maps of keywords based on co-occurrence, and it is applied for the analysis of countries, authors, and keywords in this study. While Citespace is a Java application for analyzing and visualizing co-citation networks, its primary goal is to facilitate the analysis of emerging trends in a knowledge domain, and it can be used for keyword clustering, which is helpful in summarizing the general research direction.

### Bibliometric Analysis

We selected the WOS of Thomson Reuters because it is a large collection of studies, especially those focused on biomedicine. The impact factor (IF) was obtained from the information provided by the journal citation reports (JCRs) published in 2020, and the relative research interest (RRI) was defined as the number of publications per year in a particular research field divided by the total number of publications across all fields. The H-index of articles serves as a tool to measure academic productivity, and it indicates that a researcher or a country has published at least H articles and that each article has been cited in other publications at least H times. It is widely accepted that the H-index plays an important role in evaluating the scientific research impact of a researcher or a country, especially in the medical field (15).

As practical statistical software, VOSviewer can use the links between nodes in the map to determine bibliometric characteristics, such as references, institutions, authors, and terms. Additionally, VOSviewer can analyse and predict potential trends in future research studies. The VOSviewer software was also used to extract keywords. Compared with Vosviewer, Citespace can complement the aspects in clustering of more keywords and analyse the type of cited articles and articles citing others, which will be more conducive to our understanding of research focus and the field that is related to the research topic (16).

## Results

### Contributions of Countries to Global Publications

**Figure 2:**
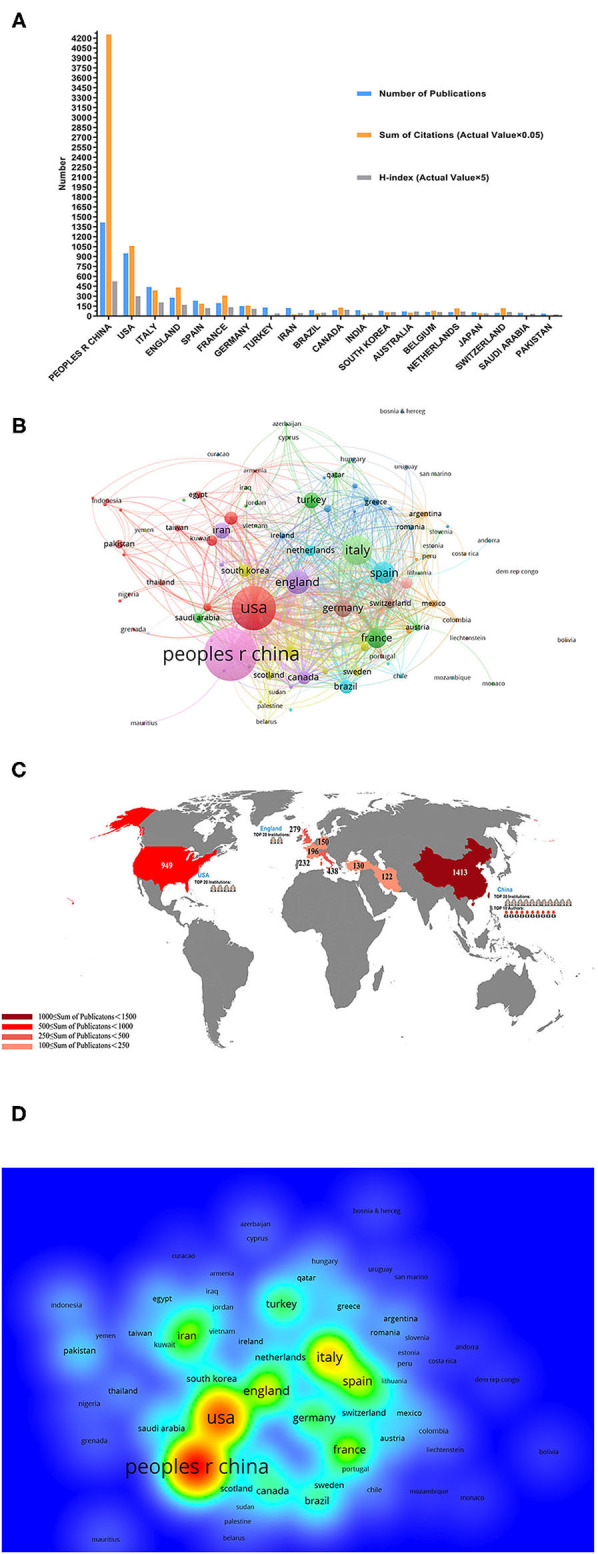
Contributions of different countries/regions to the research studies regarding coronavirus disease-2019 (COVID-19). **(A)** The number of publications, citation frequency (×0.05), and H-index (×5) of the top 20 countries or regions with the highest number of publications. **(B)** Bibliometric analysis of citations by country. Different colors indicate different clusters, and the size of circles reveals the number of citations. **(C)** The number of publications regarding COVID-19 worldwide. **(D)** Density map of countries.

In total, 4,092 articles (7,238 articles that did not meet the type requirements and 11,545 articles that were not related to the topic were excluded) published from January 2020 to December 2020 met the inclusion criteria. China ranked first in the number of publications with 1,413 articles (30.4%), which accounted for approximately one-third of the total publications, followed by the United States (USA) at 949 (20.4%) and Italy at 438 (9.42%) (Figure 2A). Figures 2B,C show that the top five countries in the field of COVID-19 with respect to the number of published articles are China, the USA, Italy, Britain, and Spain. As shown in Figure 2D, the regional correlation shows that almost all of the countries that published many articles are mainly from Europe, the USA, and Asia. China, with the largest number of publications, also ranked first globally in regard to institutions, and Chinese authors issued most of the articles.

### Citation and H-index Analysis

According to the JCRs from the WOS database, all clinical studies related to COVID-19 have been cited 150,402 times (141,101 times when self-citations were excluded), with an average citation frequency of 32.38 times per article. As the country with the largest number of publications, China accounted for 56.57% of the total citations (85,081 times [77,911 times when self-citations were excluded]) and had an H-index of 105. The number of citations from the USA was 21,203 (20,015 when self-citations were excluded), with an H-index of 60; thus, the USA ranked second among all the involved countries and districts (Figure 2A).

### Institutions With Clinical Research Publications on COVID-19

**Figure 3:**
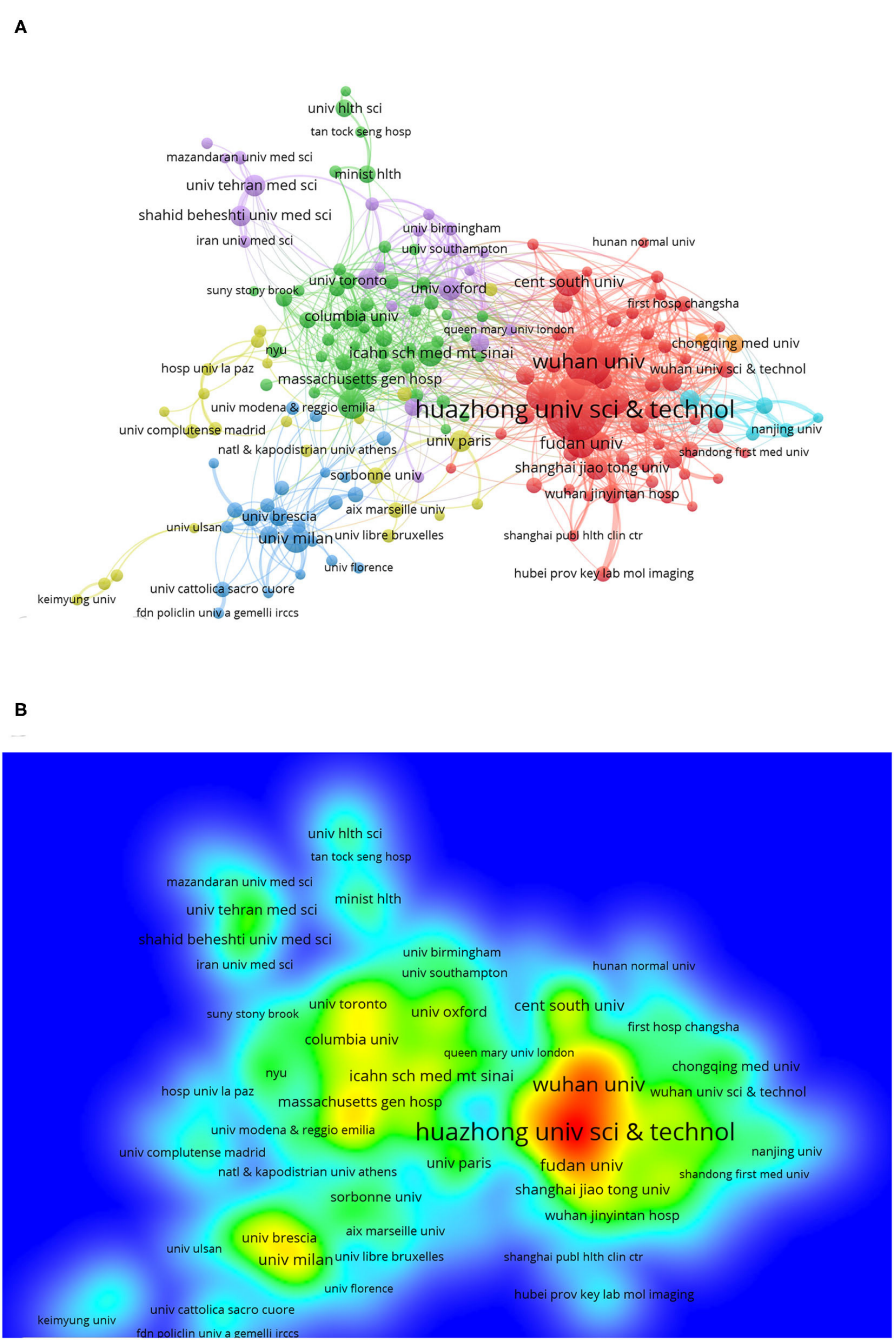
Distribution of institutions focused on COVID-19. **(A)** Bibliometric analysis of citations by the institution. Different colors indicate different clusters, and the size of circles reveals the number of publications. **(B)** Density map of institutions.

Institutions that had more than 10 publications and cited more than 20 times are included in Figure 3A. Huazhong University of Science and Technology had the most publications; the 376 articles from Huazhong University of Science and Technology have been cited 31,727 times, and we can get clear information about institutional contribution based on color (red represents more contributions, and green represents less contributions) from Figure 3B. Figure 2B shows that Huazhong University of Science and Technology, Wuhan University (213 articles), and Capital Medical University (80 articles) are the top three publishing institutions. On the list of the top 20 institutions, 11 were Chinese (mainland China and Taiwan district) institutions, 4 were institutions located in the USA, 2 were English institutions, and the other 3 institutions were in France, Iran, and Italy.

### Journals About Clinical Research Publications on COVID-19

**Figure 4:**
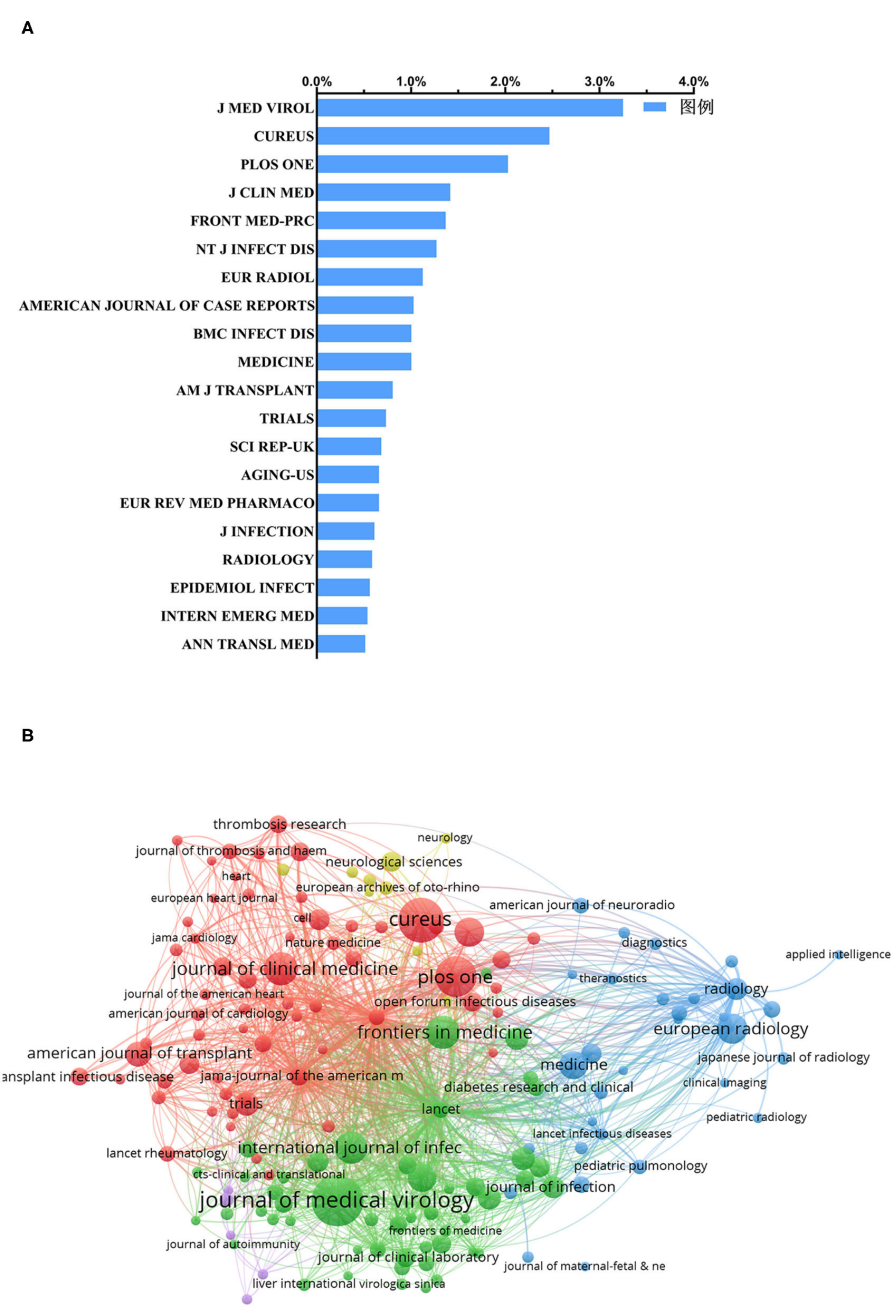
Distribution of journals focused on COVID-19. **(A)** Distribution of the top 20 journals publishing the most clinical research studies on COVID-19. **(B)** Bibliometric analysis of citations by the journal. Different colors indicate different clusters, and the size of circles reveals the number of publications.

Overall, the publications included in this analysis do not appear to be concentrated in a few journals. The total publications of the top 20 journals accounted for 22.3% of the total literature included in this analysis, while the publications of the top-ranked journal (*Journal of Medical Virology*) accounted for only 3.3% (Figure 4A). As shown in Figure 3A, the number of papers published in the *Journal of Medical Virology* (IF = 2.0218) was the highest, with 133 records, which were cited 2,787 times. Cureus (no IF) ranked second, with 101 publications that were cited 101 times. Additionally, the journal with the highest IF was Radiology (IF = 7.931) and ranked 17th, with a total of 24 publications.

### Articles About Clinical Research Publications on COVID-19

The most cited article about the use of publications on COVID-19 worldwide was *Clinical features of patients infected with 2019 novel coronavirus in Wuhan, China*, which was published by Wang, JW in China, and this article was cited 9,702 times. Unsurprisingly, among the top 10 most cited articles listed in this field, 9 came from China. It should be noted that 4 of the top 10 most cited articles were published in the special columns for COVID-19 of *The Lancet*, and 8 of them had an IF of over 20 (Table 1).

**Table 1:** Top 10 most cited studies on coronavirus disease-2019 (COVID-19).

**Title**	**Corresponding authors**	**Journal**	**IF**	**Publication year**	**Total citations**	**Article type**
Clinical features of patients infected with 2019 novel coronavirus in Wuhan, China	Wang, JW	LANCET	60.39	2020	9,702	Descriptive study
Clinical Characteristics of Coronavirus Disease 2019 in China	Zhong, N	NEW ENGLAND JOURNAL OF MEDICINE	74.699	2020	5,933	Descriptive study
Clinical Characteristics of 138 Hospitalized Patients With 2019 Novel Coronavirus-Infected Pneumonia in Wuhan, China	Peng, ZY	JAMA-JOURNAL OF THE AMERICAN MEDICAL ASSOCIATION	45.54	2020	5,494	Descriptive study
A Novel Coronavirus from Patients with Pneumonia in China, 2019	Wu, GZ	NEW ENGLAND JOURNAL OF MEDICINE	74.699	2020	5,189	Retrospective cohort study
Clinical course and risk factors for mortality of adult inpatients with COVID-19 in Wuhan, China: a retrospective cohort study	Chen, H	LANCET	60.39	2020	5,046	Retrospective cohort study
Epidemiological and clinical characteristics of 99 cases of 2019 novel coronavirus pneumonia in Wuhan, China: a descriptive study	Zhang, L	LANCET	60.39	2020	4,934	Descriptive study
A familial cluster of pneumonia associated with the 2019 novel coronavirus indicating person-to-person transmission: a study of a family cluster	Yuen, KY	LANCET	60.39	2020	2,423	Descriptive study
Clinical course and outcomes of critically ill patients with SARS-CoV-2 pneumonia in Wuhan, China: a single-centered, retrospective, observational study	Shang, Y	LANCET RESPIRATORY MEDICINE	25.094	2020	1,964	Single-centered, retrospective, observational study
Abnormal coagulation parameters are associated with poor prognosis in patients with novel coronavirus pneumonia	Sun, ZY	JOURNAL OF THROMBOSIS AND HAEMOSTASIS	4.157	2020	1,665	Retrospective cohort study
Hydroxychloroquine and azithromycin as a treatment of COVID-19: results of an open-label non-randomized clinical trial	Raoult, D	INTERNATIONAL JOURNAL OF ANTIMICROBIAL AGENTS	4.621	2020	1,475	non-randomized clinical trial

### Clinical Trials on COVID-19

The results showed that there were a total of 17 trials and that the 17 clinical trials were an independent part, which is different from the 4,092 articles extracted from WOS. Nearly 20 countries participated in the 17 trials, with 5 of the 17 included trials being from the USA. Although China is the country with the largest number of publications, it does not contribute in this regard at present. Of note, one of the trials was sponsored and conducted by more than 10 countries. With respect to research purposes, unsurprisingly, 10 of the 17 studies involved treatments. Similarly, 13 of the 17 trials mostly involved drug selection and use. Treatment-related clinical trials also covered a wide range of drugs, such as areplivir, favipiravir, povidone-iodine, and hydroxychloroquine. Of note, there are six trials in this field related to ivermectin (Supplementary Table 1).

### Analysis of Keywords About Clinical Research Publications on COVID-19

**Figure 5:**
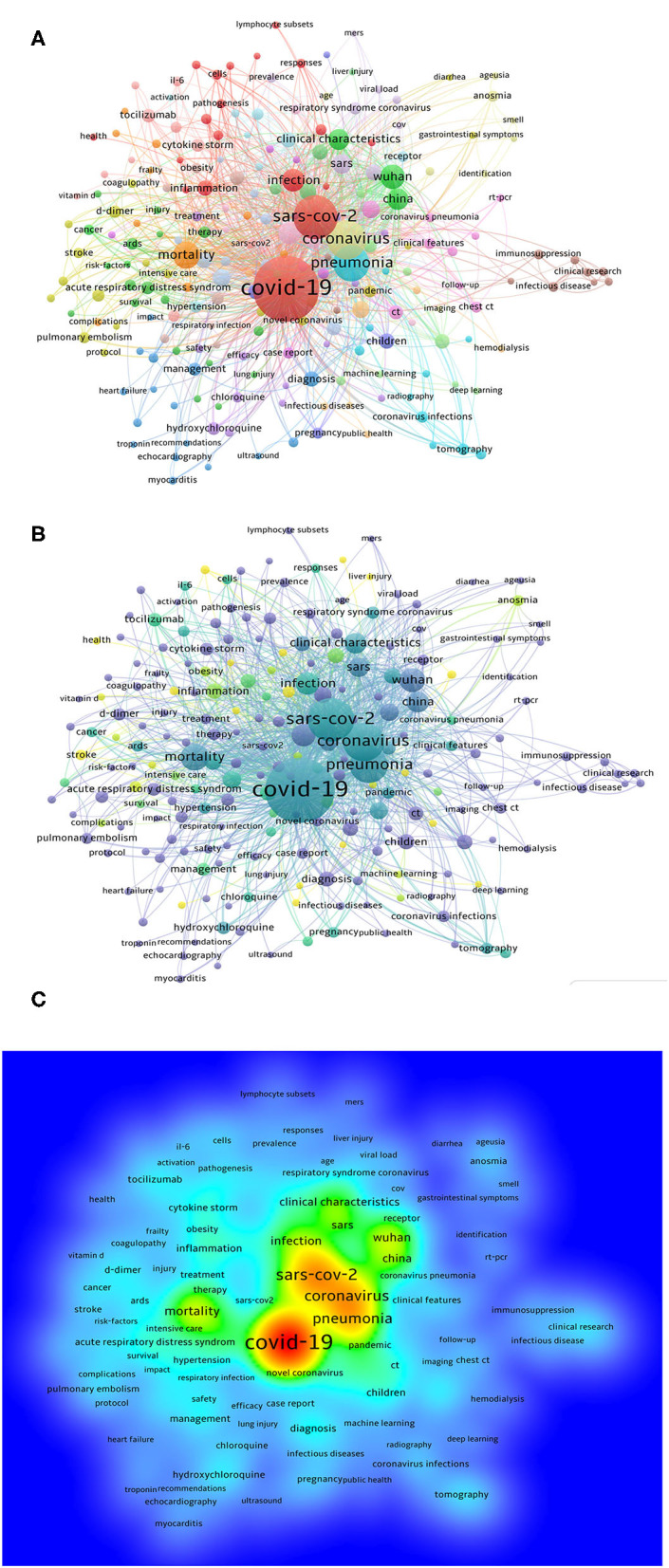
Bibliometric analysis of keywords about clinical research publications on COVID-19. **(A)** Distribution of keywords. **(B)** Network map of trend topics according to keywords used from January 2020 to June 2020. The indicator shows current publications from purple to yellow. The size of the circles represents the frequency of appearance, whereas the distance between two circles indicates their correlation. **(C)** Density map of keywords.

The keywords chosen by article authors when they submitted their manuscripts for publication were extracted with VOSviewer. We analyzed the keywords extracted from 4,092 publications. As shown in Figure 5A, 218 keywords, defined as terms that occurred more than 15 times within titles and abstracts in all articles during the analysis, are frequently mentioned, such as COVID-19 (2,367 times), SARS-CoV-2 (973 times), coronavirus (743 times), pneumonia (627 times), mortality (323 times), and Wuhan (261 times).

Detailed data on the co-occurrence of all included keywords are presented in Figure 5A. More graphical data are shown in Figure 5B. As shown in Figure 5C, VOSviewer color all keywords according to the average number of appearances of each word. In particular, the color blue indicates that the word appeared relatively early, while the color yellow indicates a more recent appearance. For example, symptoms, cytokine, comorbidities, hyperglycaemia, artificial intelligence, viral shedding, liver injury, and corticosteroids are eight of the earlier keywords that have been highlighted recently, and the two most recent keywords are pandemic and meta-analysis, which also reflects the change in the focus and the future research trend to a certain extent.

### Clustering Analysis of Types of Articles and Keywords

**Figure 6:**
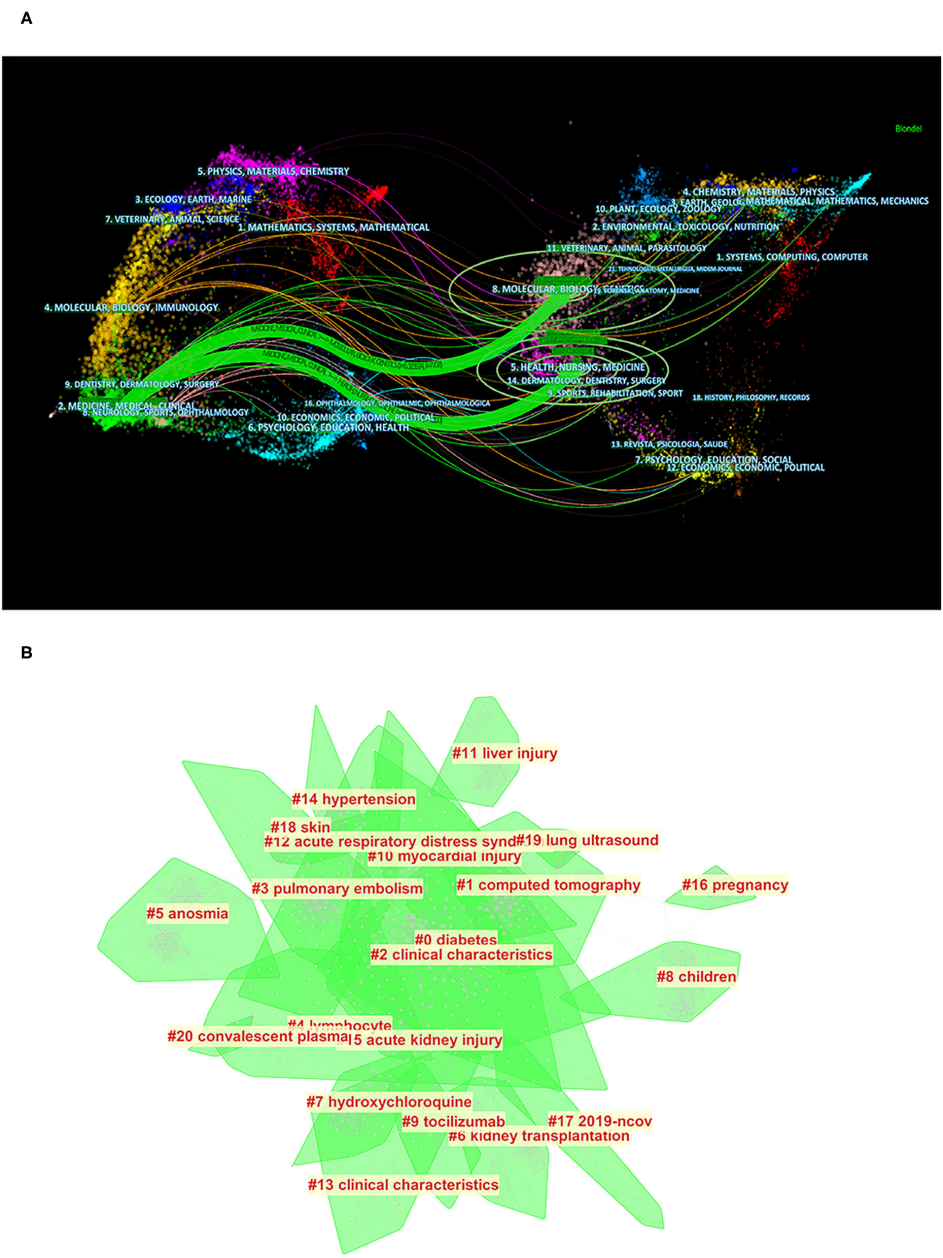
Bibliometric analysis of the clustering of types of articles and keywords. **(A)** Clustering analysis of articles being cited and articles that cited literature (distribution of cited literature and journals on the left and articles that cited literature on the right). **(B)** Clustering analysis of keywords (the order is from 0 to 20; the smaller the number, the more keywords the cluster contains, and therefore the more attention it receives).

As shown in Figure 6A, cited journals are distributed across the fields of medicine and medical and clinical research, while cited references come from two major clusters, one of which includes molecular, biology, and genetics, and the other includes healthy, nursing, and medicine. In the co-cited analysis of keywords, the top five clusters were diabetes, CT, clinical characteristics, pulmonary embolism, and lymphocytes (Figure 6B), which reflected the focus of keywords in the field on the clinical features, examination methods, test methods, and underlying diseases and complications of the disease.

## Discussion

### Research Trends in Clinical Research Publications on COVID-19

In terms of the publication volume of all countries, China ranked first, followed by the USA and Italy (Figure 2A). Similarly, Asia, North America, and Europe were also the areas hardest hit by COVID-19 (Figure 2C). We speculate that the reason for this phenomenon is related to the timing of the virus outbreak and the number of people infected with the disease. China topped the list, because it was the first country to be affected. As early as December 2019, SARS-CoV-2 was identified as the cause of the onset of an outbreak of the respiratory disease in Wuhan, Hubei province, China (17). The fact that the USA and Italy were the areas most affected by COVID-19 may explain their high number of published articles. As of April 7, 2020, Italy had the second highest number of registered cases in Europe (135,586) (18). Additionally, as of early June 2020, SARS-CoV-2 has infected more than 6.3 million people worldwide, of whom more than 1.9 million were from the USA, which also had the highest number of infections and deaths from the outbreak (19).

At present, the articles that have mainly appeared in this field have not been published in a certain type of journal (Figure 4A). The top 20 journals according to the number of published articles covered epidemiology (20), virology (21), radiology (22), emergency medicine (23), and more. It is reasonable to believe that COVID-19 has attracted the attention of a wide range of medically relevant fields, not just one discipline, which is also an indication that the solution to the COVID-19 pandemic will require the communication of various disciplines.

In terms of institutions, the top two institutions were Huazhong University of Science and Technology and Wuhan University. As the city where the COVID-19 outbreak first occurred, Wuhan has had the first-hand clinical case and clinical treatment experience (17), so the number of published articles from this city is relatively high. Although COVID-19 is globally prevalent, the top 20 publishing agencies were all from Europe and North America except for China, which is related to the advanced level of research studies in Europe and North America in the medical field, especially when COVID-19 was combined with other diseases. For example, the guidelines for tracheal intubation for patients with COVID-19 were developed by the Canadian Pediatric Anesthesia Society (18), and the guidelines for oncology treatment for patients with COVID-19 were based on the oncology guidelines in the United Kingdom and the USA (24).

Table 1 shows the detailed information on COVID-19 and the 10 most frequently cited publications. The top three cited articles, published in *The Lancet, New England Journal of Medicine*, and *JAMA—Journal of the American Medical Association* (25), described clinical manifestations in patients with COVID-19, and 4 of the top 10 articles were related to COVID-19 clinical features. Similarly, among the top 10 articles, there were 3 related to the prognoses of patients. Therefore, we have a reason to believe that the clinical manifestations, characteristics, treatments, and prognoses of COVID-19 patients are the key points of research study at present.

### Research Focused on Clinical Research Publications on COVID-19

Figure 5 shows that almost all of the cited articles are from the fields of medicine and medical and clinical research. The articles being cited are mostly from the fields of health, nursing, and medicine, as well as molecular, biology, and genetics, which indicate that the ideal solution to the COVID-19 pandemic will require the joint efforts of researchers from multiple fields such as basic biology and genetics. In particular, molecular studies have speculated on the pathogenesis of COVID-19, and angiotensin-converting enzyme 2, expressed in human vascular endothelium, respiratory epithelium, and other cell types, is thought to be a primary mechanism of SARS-CoV-2 entry and infection (26). At the same time, in the cell biology field, macrophages, as key cells in response to viral infection, can lead to the aggravation of infection. While an adaptive immune response is essential for the elimination of SARS-CoV-2, in some cases, macrophages show significant production of IL-6, suggesting that they may cause excessive inflammation in COVID-19 (27). Together, these findings also suggest that the ideal solution to the COVID-19 pandemic will require the involvement of researchers in molecular science, cytology, and other basic medicine fields.

In terms of clustering analysis of keywords, the top five keyword clusters included diabetes, CT, clinical characteristics, pulmonary embolism, and lymphocytes, and the above five clusters represented the research studies on basic diseases, clinical manifestations, examinations, complications, and tests. A study has shown that diabetes predisposes patients to a particularly severe course, with COVID-19 doubling the risk of death because of lung and heart involvement (28). As an effective test index, a reduced peripheral blood absolute lymphocyte count with an elevated neutrophil count has been a consistent observation in hospitalized patients with COVID-19 and has certain significance in the diagnosis of the disease (29). Symptoms, such as fever, cough, and fatigue (17), also indicate the possibility of SARS-CoV-2 infection. At present, the findings from chest CT and reverse transcriptase-polymerase chain reaction (RT-PCR) can complement each other in the clinical diagnosis of COVID-19. Consolidation, a reticular pattern, and a crazy-paving pattern are typical CT manifestations of COVID-19 (30). The results of this study also suggest that the prevalence of pulmonary embolism in patients with COVID-19 is close to 5% in the whole population and that pulmonary embolism seems to be associated with more extensive lung damage (31). Through clustering analysis of keywords, we have a reason to believe that the diagnosis, prevention, and prognosis of the disease are still the focus of our attention at present.

A map of all the keywords and hotspots (Figures 5A–C) was obtained and analyzed. Among the 10 latest hotspots, 8 were closely related to symptoms, underlying diseases, complications, and treatment. Surprisingly, the two most recent hotspots were pandemics and meta-analyses. Pandemic is an epidemiological term that describes the severity of the spread of infectious diseases. The outbreak of COVID-19 in China was brought to global attention and declared a pandemic by the WHO (32). The fact that the disease has crossed national boundaries in such a short period of time is sufficient to demonstrate that the problem needs to be addressed from an epidemiological perspective. Meta-analyses, with their statistical content, are important features of clinical trial evaluation because of their objectivity and systematic nature. Meta-analyses are of great significance for the integration and analysis of clinical research (33). In COVID-19 research, many studies in this area have come to the attention of the authors, especially those regarding the risk factors, treatment effects, and diagnosis of the disease (34). Meta-analyses have helped clinicians and researchers summarize problems in the field of COVID-19.

Clinical research studies address the diagnosis, treatment, prognostic prediction, and prevention of diseases, and the results of the research studies can be quickly converted to clinical applications. As a highly contagious epidemic, COVID-19 has received global attention, clinical research study is a key factor in addressing the pandemic in this filed. Clinical trials can provide highly reliable evidence. We searched clinicaltrials.gov and obtained 17 documented clinical trials (Supplementary Table 1). The results from this field showed that most of the trials were related to treatment, especially the use of drugs. Thirteen of the studies were related to specific drugs. At present, antiviral drugs, such as areplivir and favipiravir, are being used in trials. The days from the onset of fever to defervescence were shown to be positively correlated with the duration from the onset of fever to the initiation of favipiravir treatment (35). However, no specific antiviral drugs have been approved for the treatment of COVID-19 (36). Therefore, more clinical trials are needed. Hydroxychloroquine, as a treatment for malaria, has also received widespread attention. Hydroxychloroquine has been shown to have a certain effect on the treatment of patients with mild disease; however, in patients with severe COVID-19, the use of hydroxychloroquine resulted in significant worsening of clinical status because of renal dysfunction and increased need for invasive mechanical ventilation (37). The clinical use of hydroxychloroquine still needs to be studied. In trials, ivermectin has attracted the most attention as an antiviral drug with potential effects, and the dose and mechanism of action of this drug are still the focus of our study (38, 39). Based on the findings, the objective of the current clinical trials is to find a more practical drug that can inhibit the transmission of SARS-CoV-2.

This study investigated publications from the WOS database and clinical trials, and we tried to obtain objective and reliable results. However, due to the limitation of the search to studies in English and the constant updating of the database, as well as the exclusion of non-research articles, the results may differ slightly from the actual results. For more comprehensive results, databases such as Medline, Scopus, or Google Scholar could be searched in further studies.

## Conclusions

China has published the most research studies on COVID-19. In terms of clinical trials, the USA has the largest number of studies, and the diagnosis, prevention, and prognosis of the disease are still the focus of our attention at present. The overall analysis of the disease from the perspective of epidemiology and statistics will receive more attention. However, finding an effective treatment remains the focus of clinical trials. The ideal solution to the COVID-19 pandemic will require the joint efforts of all disciplines.

## Data Availability Statement

The original contributions presented in the study are included in Supplementary Material.

## Author Contributions

DMX and RQY collected, analyzed the data, and wrote the manuscript. DMX, SW, GQC, and YW designed the study and revised the manuscript.

## Conflict of Interest

The authors declare that the research was conducted in the absence of any commercial or financial relationships that could be construed as a potential conflict of interest.

## Publisher's Note

All claims expressed in this article are solely those of the authors and do not necessarily represent those of their affiliated organizations, or those of the publisher, the editors and the reviewers. Any product that may be evaluated in this article, or claim that may be made by its manufacturer, is not guaranteed or endorsed by the publisher.
