# Supplementary material for: Mapping Trends and Hotspots Regarding Clinical Research on COVID-19: A Bibliometric Analysis of Global Research
Source: Front Public Health. 2021 Aug 23;9:713487. doi: 10.3389/fpubh.2021.713487 (PMC8419357; doi:10.3389/fpubh.2021.713487)
Supplement: Supplementary file 1 [file Table_1.DOCX]

**Supplementary Table 1. Seventeen documented clinical trials related to COVID-19.**

| **Study** | **Office title** | **Country** | **Design** | **No. of patients** | **Intervention** | | **Primary outcome measurement** | **Primary purpose** | **Summary** |
| --- | --- | --- | --- | --- | --- | --- | --- | --- | --- |
|  |  |  |  |  | **Treatment group** | **Comparison group** |  |  |  |
| 1 | Open-label Randomized Multicenter Comparative Study on the Efficacy and Safety of Areplivir Film-coated Tablets (PROMOMED RUS LLC, Russia) in Patients Hospitalized With COVID-19 | Russian | Randomized,Parallel Assignment,Masking: None (Open Label) | 200 | Favipiravir | Standard of care | Clinical status improvement by WHO Ordinal Scale for Clinical Improvement (WHO-OSCI) | Treatment | This is open-labe randomized multicenter comparative Phase III study conducted in 5 medical facilities. The objective of the study is to assess the efficacy and safety of Favipiravir compared with the Standard of care (SOC) in hospitalized patients with moderate COVID-19 pneumonia. |
| 2 | A Multicenter, Adaptive, Randomized Blinded Controlled Trial of the Safety and Efficacy of Investigational Therapeutics for the Treatment of COVID-19 in Hospitalized Adults | United States, Denmark, Germany, Greece, Japan, Korea, Republic of, Mexico, Singapore, Spain, United Kingdom | Randomized,Parallel Assignment,Masking: Double (Participant, Investigator); | 1062 | Remdesivir | Placebo | Time to Recovery | Treatment | This study is an adaptive, randomized, double-blind, placebo-controlled trial to evaluate the safety and efficacy of novel therapeutic agents in hospitalized adults diagnosed with COVID-19. The study is a multicenter trial that will be conducted in up to approximately 100 sites globally. The study will compare different investigational therapeutic agents to a control arm. |
| 3 | The PATCH Trial (Prevention And Treatment of COVID-19 With Hydroxychloroquine) | United States | Randomized,Parallel Assignment,Masking: Triple (Participant, Care Provider, Investigator); | 173 | Hydroxychloroquine Sulfate 400 mg twice a day; 600 mg twice a day; 600 mg once a day | Placebo oral tablet | Time to Release From Quarantine Time; Time to Hospital Discharge; Number of Health Care Workers Who Developed SARS-COV-2 Infection | Treatment | The PATCH trial (Prevention And Treatment of COVID-19 with Hydroxychloroquine) is funded investigator-initiated trial that includes 3 cohorts. Cohort 1: a double-blind placebo controlled trial of high dose HCQ as a treatment for home bound COVID-19 positive patients; Cohort 2: a randomized study testing different doses of HCQ in hospitalized patients; Cohort 3: a double blind placebo controlled trial of low dose HCQ as a preventative medicine in health care workers. |
| 4 | A Pilot, Open-Labelled, Randomised Controlled Trial Of Povidone-Iodine Vs Essential Oil And Tap Water Gargling For COVID-19 Patients | Malaysia | Randomized,Parallel Assignment,Masking: Double (Participant, Investigator); | 20 | Povidone-Iodine,Essential oils | Tap water | Early Viral Clearance ( two successive readings of negative PCR swab) | Treatment | The purpose of this study is to assess the ability of regular gargling to eliminate severe acute respiratory syndrome coronavirus 2 (SARS-CoV-2) in the throat and nasopharynx. This 4 arms interventional study compares the effect of gargling using povidone-iodine, essential oils- based, tap water with control (no intervention) among Stage 1 coronavirus disease-2019 (COVID-19) patients. Findings from this study will provide new insight into the importance of gargling in the treatment and prevention of COVID-19. |
| 5 | Off Label Study to Evaluate the Efficacy of Hydroxychloroquine as Prophylaxis to Prevent Severe Acute Respiratory Syndrome Coronavirus 2 (SARS-CoV-2) Infection Among Health Care Workers at High Risk of Occupational Exposure to SARS-CoV-2 | United States | Non-Randomized,Parallel Assignment, Masking: None (Open Label) | 1 | Hydroxychloroquine | NE | SARS-CoV-2 Infection(PCR) | Prevention | The HCW Prophylaxis (HCWP) Study, single, open and off label intervention study. Up to 350 participants will be assigned to group that takes HCQ or group that opts to not take study medication. Participants will be UNM HEALTH SYSTEM HCW at high risk for occupational exposure to SARSCoV- 2. Study timepoints will include Day 1 screening/enrollment, 30 day, 60 day, and 90 day assessments. Questionnaires will be collected in all timepoints. |
| 6 | A Phase III Trial to Promote Recovery From Covid 19 With Combined Doxycycline and Ivermectin Along Standard Care | Bangladesh | Randomized,Parallel Assignment,Masking: Double (Participant, Investigator); | 400 | Ivermectin and Doxycycline | Standard of care | Number of Patients With Early Clinical Improvement ( by WHO and Bangladesh local guideline) | Treatment | This study has been planned to conduct an experimental clinical trial using combination of ivermectin and doxycycline for treatment of COVID 19 along with the other standard care. |
| 7 | Effectiveness of Ivermectin as add-on Therapy in Effectiveness of Ivermectin as add-on Therapy in COVID-19 Management (An Externally Controlled Pilot Trial)COVID-19Management | Iraq | allocation: N/A, Single Group Assignment, Masking: None (Open Label) | 16 | Ivermectin (IVM) |  | Number of Cured Patients(two successive readings of negative PCR swab) | Treatment | Comparing the effectiveness of Ivermectin( IVM) +Hydroxychloroquin + azithromycin (AZT) group to Hydroxychloroquin (HCQ) + azithromycin (AZT) |
| 8 | USEFULNESS of Topic Ivermectin and Carrageenan to Prevent Contagion of Covid Among Healthy People and Health Personnel | Argentina | Prospective Cohort | 229 | Combination Product: Iota carrageenan nasal spray and Ivermectin oral drops (used as buccal drops) |  | Number of Infected Subjects | Other | Estimation of the prevalence and contagiousness of undocumented novel coronavirus infections is critical for understanding the overall prevalence and pandemic potential of this disease.It is estimated that 86% of all infections were undocumented [95% credible interval (CI): 82-90%] before the 23 January 2020 travel restrictions. The transmission rate of undocumented infections per person was 55% the transmission rate of documented infections (95% CI: 46-62%), yet, because of their greater numbers, undocumented infections were the source of 79% of the documented cases. Ivermectin + Carrageenan, taking advantage of their virucidal effects, are aimed at reducing the contagion. |
| 9 | Clearing the Fog: Is HCQ Effective in Reducing COVID-19 progression-a Randomized Controlled Trial | Pakistan | Randomized,Parallel Assignment,Masking: None (Open Label) | 540 | HCQ |  | Number of Participants With Progression | Treatment | Purpose of this study is to evaluate efficacy of hydroxychloroquine (HCQ) in reducing progression of Corona Virus Disease 2019 (COVID - 19) and achieving viral clearance. |
| 10 | Use of Ivermectin as a Prophylactic Option in Asymptomatic Family Close Contact for Patient With COVID-19 | Egypt | Randomized,Sequential Assignment,Masking: None (Open Label) | 340 | Ivermectin Tablets | NE | Development of Symptoms | Prevention | asymptomatic family close contact of confirmed COVID-19 patient will receive prophylactic ivermectin and will be followed up for 14 days for any symptoms & diagnosis of COVID-19 |
| 11 | The Protocol of Evaluation of Safety and Efficiency of Method of Exosome Inhalation in SARS-CoV-2 Associated Two-Sided Pneumonia | Russian | Randomized,Parallel Assignment,Masking: Double (Participant, Care Provider); | 30 | EXO 1 inhalation, EXO 2 inhalation | Placebo inhalation | Number of Participants with Non-serious and Serious Adverse Events During Trial and Inhalation Procedure | Treatment | This protocol has been developed based on the literature, information about the ongoing tests NCT04276987 (A Pilot Clinical Study on Inhalation of Mesenchymal Stem Cells Exosomes Treating Severe Novel Coronavirus Pneumonia) and NCT04384445 (Organicell Flow for Patients With COVID-19), Patent No 271036826 of 2019. "A method for obtaining and concentrating microRNA-containing exosomal multi-potent mesenchymal-stromal cells for use in cosmetic and pharmaceutical products to stimulate regenerative processes and slow down aging. |
| 12 | Convalescent Plasma in the Treatment of COVID 19 | United States | allocation: N/A, Single Group Assignment, Masking: None (Open Label) | 48 | Convalescent Plasma | NE | Mortality within 28 days,Viral Load, Serum Antibody Titers | Treatment | The purpose of this study is to collect blood from previously COVID-19 infected persons who have recovered and use it as a treatment for those who are currently sick with a severe or life-threatening COVID-19 infection. |
| 13 | Prolonged Low Doses of Methylprednisolone for Patients With COVID-19 Severe Acute Respiratory Syndrome | Italy | Prospective Cohort | 173 | Methylprednisolone | standard care | In-hospital Death Within 28 Days , Admission to Intensive Care Unit (ICU), Endotracheal Intubation | Other | The main objective of this multi-centre observational trial is to analyse the association of low dose prolonged infusion of methylprednisolone (MP) for patients with severe acute respiratory syndrome with composite primary end-point (ICU referral, need for intubation, in-hospital death at day 28). |
| 14 | Ivermectin, Aspirin, Dexamethasone and Enoxaparin as Treatment of Covid 19 | Argentina | Prospective Cohort | 167 | Drug: Ivermectin 5 MG/ML oral solution, Aspirin 250 mg tablets, Other: Ivermectin 5 mg/mL oral solution, Dexamethasone 4-mg injection, Aspirin 250 mg tablets Other: Ivermectin 5 MG/ML oral solution, Dexamethasone 4-mg injection, Enoxaparin injection. Inpatient treatment with mechanical ventilation in ICU. | NE | Mortality,Drug Dose Adjustment, Adverse Events, ICU-treated Patients | Other | The associated use of Ivermectin, aspirin, dexamethasone, and enoxaparin (in different combinations and doses) will reduce the impact of COVID infection 19, the need of admission to the intensive care unit, and mortality. |
| 15 | Observational Open Study of Polyoxidonium in Hospitalized Patients With COVID-19 | Russian | Prospective Cohort | 81 | Polyoxidonium |  | Clinical Status of the Patient | Other | The study is designed as an open observational non-comparative study of Polyoxidonium®, lyophilizate for solution for injections and topical application, 12 mg in hospitalized patients with coronavirus disease (COVID-19). |
| 16 | Bioequivalence Study of Favipiravir 200 mg Film Tablet (Novelfarma, Turkey) Under Fasting Conditions | Turkey | Randomized,Crossover Assignment, Masking: None (Open Label) | 30 | Drug: FAVIRA 200 mg Film Tablet, Drug: AVIGAN 200 mg Film Tablets, |  | AUC0-tlast, Favipiravir Cmax | Other | A single dose of Reference product containing 200 mg favipiravir and a single dose of Test product containing 200 mg favipiravir or vice versa; administered with 240 mL of water at room temperature, in each period under fasting conditions with current pandemic precautions. |
| 17 | Pilot Study to Evaluate the Potential of Ivermectin to Reduce COVID-19 Transmission | Spain | Randomized,Parallel Assignment,Masking: Double (Participant, Investigator); | 24 | Ivermectin | Placebo | Proportion of Patients with a Positive SARS-CoV-2 PCR | Treatment | SAINT is a double-blind, randomized controlled trial with two parallel groups that evaluates the efficacy of ivermectin in reducing nasal viral carriage at seven days after treatment in SARS-CoV-2 infected patients who are at low risk of progression to severe disease. The trial is currently planned at a single center in Navarra. |
